# Chemical warfare agent simulants for human volunteer trials of emergency decontamination: A systematic review

**DOI:** 10.1002/jat.3527

**Published:** 2017-10-09

**Authors:** Thomas James, Stacey Wyke, Tim Marczylo, Samuel Collins, Tom Gaulton, Kerry Foxall, Richard Amlôt, Raquel Duarte‐Davidson

**Affiliations:** ^1^ Chemical and Environmental Effects Department Centre for Radiation, Chemicals and Environmental Hazards (CRCE), Public Health England Chilton OX11 0RQ UK; ^2^ Toxicology Department Centre for Radiation, Chemicals and Environmental Hazards (CRCE), Public Health England Chilton OX11 0RQ UK; ^3^ Emergency Response Department Science & Technology, Public Health England Porton Down, Salisbury Wiltshire SP4 0JG UK

**Keywords:** chemical warfare agent, decontamination, emergency, human volunteer trials, simulant

## Abstract

Incidents involving the release of chemical agents can pose significant risks to public health. In such an event, emergency decontamination of affected casualties may need to be undertaken to reduce injury and possible loss of life. To ensure these methods are effective, human volunteer trials (HVTs) of decontamination protocols, using simulant contaminants, have been conducted. Simulants must be used to mimic the physicochemical properties of more harmful chemicals, while remaining non‐toxic at the dose applied. This review focuses on studies that employed chemical warfare agent simulants in decontamination contexts, to identify those simulants most suitable for use in HVTs of emergency decontamination. Twenty‐two simulants were identified, of which 17 were determined unsuitable for use in HVTs. The remaining simulants (n = 5) were further scrutinized for potential suitability according to toxicity, physicochemical properties and similarities to their equivalent toxic counterparts. Three suitable simulants, for use in HVTs were identified; methyl salicylate (simulant for sulphur mustard), diethyl malonate (simulant for soman) and malathion (simulant for VX or toxic industrial chemicals). All have been safely used in previous HVTs, and have a range of physicochemical properties that would allow useful inference to more toxic chemicals when employed in future studies of emergency decontamination systems.

## Abstract 2

To conduct human volunteer trials on the decontamination of chemical warfare agents, simulant chemicals that mimic the properties of the live agents must be used. For the first time, this review identifies previously used chemical simulants and assesses their suitability according to toxicity, physicochemical properties and similarity to their equivalent toxic counterparts.

## 1 INTRODUCTION

A chemical incident is traditionally defined as an unexpected or uncontrolled release of a chemical from its containment. While rare, incidents involving the exposure of large numbers of people to chemical contaminants have taken place (WHO, 1999, 2002). Chemical incidents may involve the accidental or deliberate release of a chemical contaminant, and the majority of incidents involve an acute release, accompanied by a rapidly rising exposure risk (WHO, 2002). Chemical incidents may be small or large in scale, and can give rise to multiple primary or secondary chemical casualties and fatalities (Baker, 2005; Duarte‐Davidson, Orford, Wyke, et al., 2014).

In a chemical incident, emergency decontamination of affected casualties needs to be undertaken to reduce injury and possible loss of life. In a real incident, decontamination must protect against potential highly toxic and hazardous chemicals, such as toxic industrial chemicals (TICs) and chemical warfare agents (CWAs) (Balali‐Mood & Balali‐Mood, 2008; Brennan, Waeckerle, Sharp, & Lillibridge, 1999; Duarte‐Davidson et al., 2014). TICs are defined as any substances (gas, liquid or solid) that are produced, stored, transported and widely used by industry and can cause harm to human health or the environment when not properly contained. They possess chemical hazards (e.g., as carcinogens or corrosives) and/or physical hazards (e.g., flammable or explosive properties). TICs are normally produced in large quantities, which differentiate them from highly toxic speciality chemicals that are produced in only limited volumes (‘Toxic Industrial Chemicals’, 2002). There are lists of TICs available in the public domain (Occupational Safety and Health Administration). CWAs are highly toxic synthetic chemicals that can be dispersed as a gas, liquid (including aerosols) or adsorbed on to particles to become a powder. CWAs have either lethal or incapacitating effects on humans, and differ from explosive chemicals where the destructive effects are localized and caused by shear force. There are thousands of toxic substances, but only a few are considered CWAs based on their characteristics, such as high toxicity, rapid action and persistency (Ganesan, Raza, & Vijayaraghavan, 2010; Technical Secretariat of Organization for Prohibition of Chemical Weapons, 1997). CWAs are generally classified according to the physiological effects on humans and include nerve agents, vesicants (blistering agents), blood agents (cyanogenic agents), choking agents (pulmonary agents), riot‐control agents (tear gases), psychomimetic agents and toxins (Occupational Safety and Health Administration).

Studies to optimize the effectiveness of emergency decontamination processes using human volunteers must, for ethical reasons, use simulants to mimic the physicochemical properties of more harmful chemicals, while remaining non‐toxic at the dose applied. A simulant is a compound that can mimic the behaviour of the chemical of interest (e.g., has similar physicochemical properties) or is a functional analogue of a more harmful chemical (Jenkins, Buchanan, Merriweather, et al., 1994), and can be used in human volunteer trials (HVTs) with minimal risk (Amlôt, Larner, Matar, et al., 2010; Josse, Comas, Bui‐Tho, et al., 2011; Larner, Matar, Riddle, et al., 2007; Ribordy, Rocksén, Dellgar, et al., 2012; Torngren, Persson, Ljungquist, et al., 1998).

While there are a range of simulants reported in the literature (in vitro, in vivo and HVTs of decontamination studies), it is often difficult to identify simulants that adequately represent the diverse physicochemical properties and physiological effects of TICs and CWAs (Lavoie, Srinivasan, & Nagarajan, 2011) and how they behave in the environment. When considering environmental fate for TICs and CWAs, additional physicochemical properties that would need to be considered include: hydrolysis, sorption, bioavailability and volatilization (Bartelt‐Hunt, Knappe, & Barlaz, 2008).

To our knowledge, a review of chemical simulants suitable for use in HVTs has not been performed. The aim of this study was to conduct a systematic review of the literature to identify chemical simulants that have previously been used in both in vivo and in vitro assessments of decontamination processes, to identify a simulant or simulants suitable for use in HVTs of emergency decontamination procedures. The suitabilities of the identified simulants were evaluated using a matrix that considered factors such as relative toxicity, biological half‐life, persistence (vapour pressure), water solubility, partition coefficient (*K*_ow_) and physicochemical similarity to their corresponding TIC or CWA.

## 2 METHODOLOGY

### 2.1 Search strategy

Ovid (accessing the MedLine database), Scopus, Google Scholar (grey literature source) and Web of Science were accessed. The databases were searched for articles, papers and reports with no restrictions on date of publication using keywords and search terms. See Supporting information (Table SA) for full search terms used. Keyword sections in articles relating to simulants were compared and common themed words applied. Synonyms of keywords, and wildcard searches (allowing for inclusion of word variation) were applied to broaden the results. Searches were conducted between June and October 2016.

### 2.2 Inclusion criteria and literature screening

Inclusion criteria to focus on relevant papers included:

- *Language*: All articles must be in English due to the lack of time and resources that would be required for the translation of papers.
- *Publication status*: All types of publication, including grey literature (letters, articles, PhD theses, internal reports, evaluations and working papers) were included in the review.
- *Range of search*: The fields of the publication that were included in the search strategy were the title, abstract and keywords. If the use of simulants was not initially obvious from the title or the abstract, the paper was omitted.
- *Simulant specificity*: All papers had to contain references to simulant quantity and specific use. These could include papers referring to qualitative or quantitative studies as long as the simulant's unit of measurement (including those described by the qualitative term ‘concentrated‘) were included and that the simulant was a suitable simulant (based on physicochemical properties) for a TIC and/or CWA.

**Figure 1:**
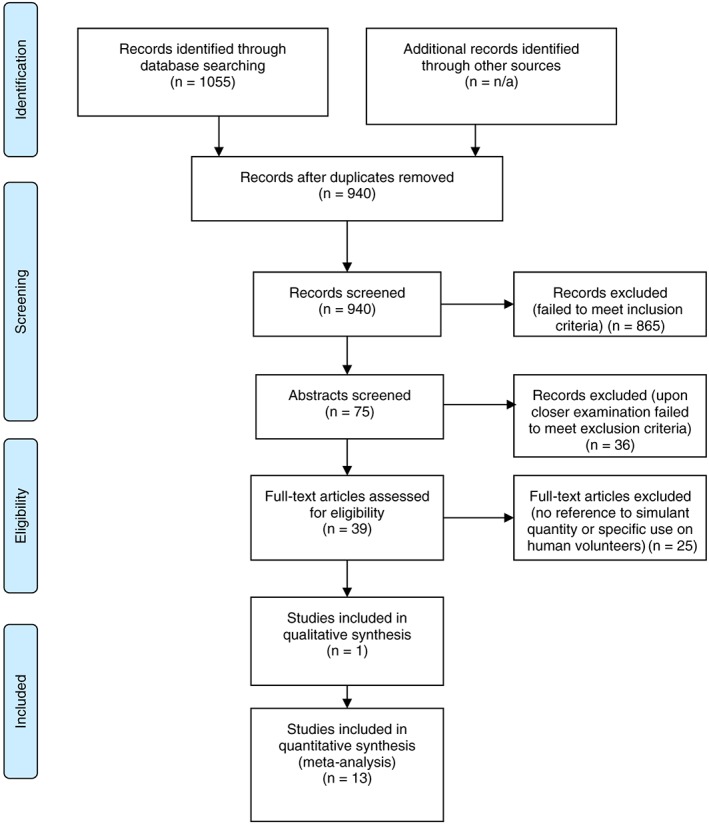
PRISMA flow diagram outlining the systematic literature review process [Colour figure can be viewed at wileyonlinelibrary.com]

Initially, search terms were applied for all fields (title, abstract, keywords), and then the search result was narrowed down through stages of manual screening that included assessing relevancy based on the title, the abstract and then the full text (Figure 1).

### 2.3 Criteria for selecting suitable simulants for human volunteer trials

#### 2.3.1 Toxicity

The toxicity of a selected simulant will depend on the dose used in HVTs. Simulants identified from studies that involved human decontamination have already been approved for use on humans, so have low toxicity at the doses used, are non‐corrosive, non‐carcinogenic, non‐mutagenic and non‐reprotoxic. Any simulants identified in the literature search, particularly those not previously used in human studies were assessed for toxicity based on data from MSDS, the Pubchem database (U.S. National Library of Medicine, 2016), the Globally Harmonized System of Classification and Labelling of Chemicals (GHS) risk phrases (Health and Safety Executive, 2017), and where applicable, the National Poisons Information Service's TOXBASE database (National Poison's Information Service, 2017).

#### 2.3.2 Persistence

Simulants should be relatively stable and persistent, i.e., they should be stable at a range of temperatures and light conditions consistent with conducting a decontamination study in human volunteers. If bioavailable following dermal application then the half‐life should be appropriate to assess uptake through 24 hour urine collection (half‐life should not be too long and urinary excretion should be the dominant route). Simulants should also have a low vapour pressure, i.e., should remain in/on the body long enough to be sampled and detected by relatively routine analytical methods. To assess persistence, the relative vapour pressure of each simulant was determined from reliable scientific literature. The lower the vapour pressure, the more persistent the simulant and the less likely it would be to evaporate prematurely. Indications of volatility (likely/unlikely to volatilise) were obtained from the UK Recovery Handbook for Chemical Incidents (Wyke, Brooke, Dobney, Baker, & Murray, 2012).

#### 2.3.3 Evaluation criteria

Potentially suitable simulants were shortlisted and evaluated according to their physicochemical properties, including the relative toxicity of the compound, stability within the human body (biological half‐life), vapour pressure and water solubility.

#### 2.3.4 Simulant suitability

Simulants were objectively evaluated according to a suitability matrix (Table 2) with criterion for suitability colour coded; red (indicating limited suitability), yellow (indicating moderate suitability) and green (indicating good suitability). The suitability colours are qualitative indications of how suitable the identified simulants may be for use in HVT studies. The most suitable simulant identified in this review may not be the best to use in every study, as every study is contextually dependent.

### 2.4 Bias

Bias was minimized by using keywords to search multiple databases and specific search criteria to refine the identified literature. All identified literature was assessed in the same manner to reduce bias in line with the PRISMA methodology (Moher, Liberati, Tetzlaff, et al., 2009), ensuring a transparent and systematic approach to the reviewing process.

## 3 RESULTS

Peer reviewed papers, scientific reports and literature (*n* = 1055) were retrieved from all searched databases and reduced to 940 following removal of duplicates. After an initial assessment (review of title and abstract), 865 papers were discounted according to the inclusion criteria (Figure 1). Twenty‐two of these excluded papers referred to ‘simulations‘of in vitro decontamination procedures using CWAs and as such did not use simulants.

While CWAs were specifically searched for, a large proportion of the literature focused on pesticides and their involvement in Chemical, Biological, Radiological and Nuclear incidents. Toxic industrial chemicals can be just as, if not more of a risk to the public than CWAs as they are manufactured in large volumes, stored, transported and used throughout the world. This study, however, is limited by the paucity of published information on TIC simulants. The majority of chemicals identified as potential TIC simulants are nerve agent simulants that commonly resemble the structure of organophosphate compounds and pesticides, such as malathion. These simulants were, however, included in both TIC‐ and CWA‐related papers, and were therefore relevant to include in this review. Of the 75 remaining papers, 36 were omitted after reviewing the abstracts as a simulant was not specifically referenced or the paper focused on the analysis of TICs and CWAs without the use of simulants.

The full texts of 39 papers were reviewed, of which 14 met all inclusion criteria. A total of 22 different simulants were identified and consideration was given to all. An assessment was made based on the toxicity data available from Sigma‐Aldrich (Singapore) material safety data sheets (MSDS), and toxicity data published in the literature amalgamated on the PubChem Compound database. Owing to reported adverse health effects and their unsuitability for use in HVTs, 17 simulants were deemed too toxic and were excluded from further assessment (Supporting information, Table SB). These discounted simulants included sulphur‐containing compounds [2‐(chloroethyl)phenyl sulphide, tetrahydrothiophene] and phosphorous containing compounds (paraoxon, *O*,*S*‐diethyl methylphosphonothioate), which can be highly toxic and potentially mutagenic (Abel, Boulware, Fields, et al., 2013; Boulware, Fields, McIvor, et al., 2012; Powell, Boulware, Thames, Vasquez, & MacLeod, 2010).

Some compounds were not initially regarded as ‘too toxic’ for human use. For example, parathion and its metabolite paraoxon were identified as potential simulants. However, exposure to parathion (and its subsequent metabolite paraoxon) can result in headaches, nausea, respiratory depression, seizures and significant and irreversible effects arising from the inhibition of acetylcholinesterase (Edwards, Yedjou, & Tchounwou, 2013; Reigart, 2013; Satar, Tap, & Ay, 2015). Therefore, parathion and paraoxon were eliminated as potential simulants for HVTs. However, malathion, the other organophosphate considered as a simulant, has a much lower order of toxicity after dermal exposure and so was not eliminated from consideration.

Tetrahydrothiophene was another potential candidate due to its relatively low toxicity; however, it also has an intensely unpleasant odour (Tetrahydrothiophene [MAK Value Documentation, 2010a], 2012). As the safety of volunteers in HVTs is paramount, anything that could cause chemical distress (such as tetrahydrothiophene's pungent odour) would be deemed unsuitable for use.

Potentially suitable simulants (*n* = 5) were identified (Table 1) and objectively evaluated (Table 2) with criterion for suitability colour coded; red (indicating limited suitability), yellow (indicating moderate suitability) and green (indicating good suitability), as a qualitative indicator for the potential for use of these shortlisted simulants in HVT studies. The actual suitability of a simulant will vary and depend on the study design and method of application.

**Table 1:** Potentially suitable simulants for human volunteer trials

Simulant used (equivalent toxic chemical)	Type of study	Summary of the study	Reference
Methyl salicylate (MeS) –(sulphur mustard)	In vitro	Simulation of vapour exposure following the release of MeS from clothing.	Feldman, 2010
		Qualitative study on the efficacy of temporary peelable coatings as a protective barrier against CWA simulants.	Gazi & Mitchell, 2012
		Preliminary evaluation of military, commercial and novel skin decontamination products.	Matar, Guerreiro, Piletsky, Price, & Chilcott, 2016
		Quantitative assessment of the absorption of CWA simulants in human skin.	Moody, Akram, Dickson, & Chu, 2007
		Simulation to show the usefulness of hair analysis for the detection of a CWA simulant, after vapour exposure.	Spiandore, Piram, Lacoste, Josse, & Doumenq, 2014
		In vitro study to determine the percutaneous absorption of sulphur mustard by applying MeS to pig skin.	Riviere, Smith, Budsaba, et al., 2001
		In vitro study on the efficacy of combined decontamination methods on decontamination efficacy of hair exposed to MeS vapours.	Spiandore, Piram, Lacoste, et al., 2016
		An in vitro study (using skin mounted on diffusion cells) into the effects of hydrodynamics, detergents and delays on the effectiveness of the ladder pipe decontamination system.	Matar, Atkinson, Kansagra, et al., 2014
	Human trial	Human trial testing the efficacy and functionality of the environment within a mass decontamination unit when contaminating humans with CWA simulants.	Ribordy et al., 2012
		Evaluation of the efficacy of a decontamination station following exposure of volunteers to ethyl lactate and MeS, simulants of sarin and mustard respectively.	Torngren et al., 1998
		Human volunteer trial assessing an optimised decontamination protocol as part of the ORCHIDS project.	Larner et al., 2007
	In silico	Use of cheminformatics to determine suitable CWA simulant choice.	Lavoie et al., 2011
Diethyl malonate (DM) – (soman)	In vitro	Qualitative determination of the effect of wet decontamination on skin hydration, and subsequent issues with decontamination of CWA simulant diethylmalonate.	Loke et al., 1999
		Simulation of soman decontamination using diethyl malonate and a showering method.	Reifenrath, Mershon, Brinkley, et al., 1984
	In silico	Use of cheminformatics to determine suitable CWA simulant choice.	Lavoie et al., 2011
Ethyl lactate (EL) – (chlorine/ Sarin)	Human trial	Evaluating the impact of environmental factors on decontamination within a mass decontamination unit when contaminating humans with CWA simulants.	Ribordy et al., 2012
		Evaluation of the efficacy of a decontamination station following exposure of volunteers to EL and MeS, simulants of sarin and sulphur mustard respectively.	Torngren et al., 1998
Malathion (MT) – (organophosphate TIC/VX)	In vitro	Quantitative assessment of absorption of CWA simulants in human skin.	Moody et al., 2007
1,3‐dichloropropane (DCP) – (sulphur mustard)	In vitro	Comparative study of breakthrough times of protective clothing, with sulphur mustard and a simulant, DCP.	Singh et al., 2000

**Table 2:** Evaluation of shortlisted simulants for human volunteer trials (*n* = 5)

		Criteria				
Simulant	Equivalent toxic chemical	Toxicity data (suitability for human trials)	Persistence (vapour pressure)	Biological half‐life	Water solubility (g/L at 25°C)	Summary
Methyl salicylate (MeS)	Sulphur mustard	LD_50_ dermal, rabbit, >5000 mg kg^−1^ LD_50_ oral, human adult, 500 mg kg^−1^	MeS = 6 Pa at 20°C Sulphur mustard = 9.6 Pa at 20°C likely to volatilise	Between 40 min and 30 h^a^	MeS = 0.7 Sulphur mustard 0.65 low water solubility	MeS has a similar persistence to sulphur mustard, with a medium vapour pressure, indicating it is likely to volatilise MeS has a low water solubility, similar to sulphur mustard but is miscible in diethyl ether, ethanol and acetone
Diethyl malonate (DM)	Soman	LD_50_ dermal, rabbit, 16 880 mg kg^−1^ (may cause gastrointestinal irritation if ingested)	DM = 36 Pa at 25°C Soma*n* = 54.7 Pa at 25°C likely to volatilise	No data	DM = 20 Soman = 21 low water solubility	DM has similar water solubility to soman, but slightly different persistence and volatility
Ethyl lactate (EL)	ChlorineSarin	LD_50_ dermal, rabbit, >5000 mg kg^−1^	EL = 300 Pa at 20 °C Sarin = 381 Pa at 25°C high vapour pressure likely to volatilise	No data	EL = 100 Sarin = 1000 water soluble	EL has a similar persistence and volatility to sarin, but there is a large difference in water solubility
Malathion	Organophosphate TIC/VX	LD_50_ dermal, rats, >4000 mg kg^−1^	Malathion = 0.005 Pa at 30 °C VX = 0.117 Pa at 25°C very low vapour pressure unlikely to volatilise	80% excreted in urine in 24 h	Malathion = 0.143 VX = 30 low water solubility	Malathion has a lower volatility than VX and a low water solubility, but is miscible in most organic solvents Malathion is very similar to other organophosphate compounds
1,3‐dichloropropane(DCP)	Sulphur mustard	LD_50_ dermal, rats, >2000 mg kg^−1^ (extremely volatile and harmful to respiratory system)	DCP = 2400 Pa at 20°C Sulphur mustard = 9.3 Pa at 20°C very high vapour pressure. Highly likely to volatilise	No data	DCP = 3 Sulphur mustard=0.65 low water solubility	DCP has limited similarity to suphur mustard aside from chemical structure (size and shape)

Green = good suitability; yellow = moderate suitability; red = limited suitability.

a Biological half‐life based on metabolism of aspirin, which has a similar metabolic pathway to MeS.

## 4 DISCUSSION

The aim of this study was to undertake a systematic literature review to identify chemical simulants that have previously been used in in vivo and in vitro assessments of decontamination processes, with the goal of identifying which simulant(s) would be most suitable for use in HVTs of emergency decontamination processes. The suitability of the shortlisted simulants was evaluated using a matrix that considered relative toxicity, biological half‐life, persistence (vapour pressure), water solubility, partition coefficient (*K*_ow_) and physicochemical similarity to their corresponding TIC or CWA.

Ultimately, the suitability of a simulant for use in HVTs will be dependent on the toxicity of the chemical. However, there is an inherent problem when defining relative toxicity values for simulants. Few of these chemicals have human commercial purposes, and as a result comprehensive and harmonized toxicity values are not available. The toxicology data that are available are limited in that mixed routes of exposure cannot be accurately compared; neither can data from studies conducted on different animals. Without comprehensive simultaneous toxicity studies on humans (which are unethical), relative toxicology data are limited to the provided values. Furthermore, toxicity data available for these chemicals is often only consistently provided as LD_50_ (lethal dose to 50% of a population) from animal studies and so is only used as an indicator of the suspected toxicity in HVT as simulants may demonstrate marked interspecies and inter‐individual variability in toxicity and in HVTs we want to avoid toxicity to any of the volunteers which may occur at concentrations significantly lower than the LD_50_.

### 4.1 1,3‐dichloropropane as a simulant for sulphur mustard

1,3‐Dichloropropane (DCP), although used in one study (Table 1) is extremely volatile. DCP vapours are known to cause respiratory distress. With LC_50_ inhalation levels as low as 2000 ppm h^−1^ (in rats and mice) (Smyth, Carpenter, Weil, et al., 1969), the inhalational risk to human volunteers would be too great, and for these reasons DCP was excluded.

### 4.2 Ethyl lactate as a simulant for chlorine or sarin

Ethyl lactate (EL) has been reported as an effective simulant for chlorine and sarin; however, EL is highly volatile and has low persistence, which makes sampling (and subsequent analysis) during a HVT challenging and potentially inaccurate – there is a risk that during a HVT a large proportion of the applied simulant could evaporate off the subject before and during any decontamination procedures. This could result in a false positive, indicating that the decontamination methods being tested are more effective than they actually are. If high concentrations of EL are present in the air there is also a potential respiratory risk to consider (the reported LC_50_ (inhalation) in rats is approximately 5400 mg m^−3^) (Bingham, Cohrssen, & Powell, 2001), which would significantly reduce the suitability of EL as a simulant for use in HVTs. The suitability for using EL will, however, depend on the research question being addressed and the study design; for example, EL may be more suitable for testing the inhalational risk associated with decontamination processes for a sarin‐like contaminant.

### 4.3 Diethyl malonate as a simulant for soman

Diethyl malonate has been identified as a potentially suitable simulant to mimic the behaviour of soman, as it has similar water solubility but slightly different persistence and volatility. However, there is also a risk that dermal absorption of diethyl malonate may be significantly increased during decontamination procedures. Loke, U, Lau, et al. (1999) suggested that the enhancement effect was attributed to either the spreading of the chemical over the skin during washing, or the transient skin hydration during washing, leading to a decrease in skin barrier properties. However, as diethyl malonate is metabolized into a relatively non‐hazardous compound (malonic acid) (Opdyke, 1975), the simulant itself has a relatively low dermal toxicity. Diethyl malonate was also identified as the lowest toxicity (based on rat oral data) simulant for soman in terms of potential exposure after volatilization (Bartelt‐Hunt et al., 2008). However, gastrointestinal irritation can occur through ingestion and could pose an issue when applying the simulant to human volunteers, meaning greater consideration should be given to application methodology to avoid accidental ingestion.

### 4.4 Malathion as a simulant for organophosphate toxic industrial chemicals and VX

One of the more suitable simulants identified from the literature for HVTs of emergency decontamination was malathion. Malathion is an organophosphate insecticide best known as a chemical used for the treatment of lice (pediculosis). It is present in some head lice treatment shampoos commonly used with children, and left on the hair and scalp for between 8 and 12 hours, indicating a suitably low projected human dermal toxicity value. The dermal LD_50_ of malathion in rats is >4000 mg kg^−1^ (Gallo & Lawryk, 1991), therefore, if malathion was applied to a 70 kg human, the potential total dermal LD_50_ would be a dose of about 280 g (a difficult dermal dose to achieve). Malathion is persistent, has low vapour pressure and volatility (i.e., it does not off‐gas), which makes malathion a good candidate as a simulant for TICs and VX. Malathion is also one of the least toxic organophosphate insecticides available and therefore can be used to mimic exposure to more toxic organophosphate pesticides, i.e., TICs. None the less, even with a low relative toxicity, obtaining ethical approval to use an organophosphate for HVTs may be a limiting factor. In recent years, there has been considerable controversy about the scientific value and ethical acceptability of studies involving experimental exposure of human volunteers to low doses of pesticides, in the context of regulatory risk assessment (London, Coggon, Moretto, et al., 2010). Furthermore, malathion has recently been classified as ‘probably carcinogenic to humans (Group 2A)’ (International Agency for Research on Cancer, 2015), which further calls into question ethical approval. It might be possible to obtain approval for a formulation containing malathion (e.g., headlice shampoo); however, the physicochemical properties of the formulation may alter the suitability of malathion as a simulant.

### 4.5 Methyl salicylate as a simulant for sulphur mustard

Methyl salicylate (MeS) was the most commonly used simulant with the most data available in the literature (*n* = 12 papers), along with validated analytical methods and established safety for use in HVTs. MeS is of similar persistence, volatility and water solubility to sulphur mustard. While MeS is likely to volatilise it is recoverable and has been used for several human volunteer decontamination studies (Larner et al., 2007; Ribordy et al., 2012; Torngren et al., 1998). Furthermore, previous literature has determined that MeS is the least toxic simulant when mimicking the adsorption/desorption of sulphur mustard in the environment (Bartelt‐Hunt et al., 2008). The oral LD_50_ of MeS in humans is 500 mg kg^−1^ whereas the dermal LD_50_ in rabbits is >5000 mg kg^−1^ indicating poor dermal uptake. The abundance of literature that reports using MeS as a simulant also leads to greater availability of optimized and validated methods of simulant application, recovery and analysis, and a clearer indication of the suitable dose for HVTs of emergency decontamination. MeS is suitably persistent to be measureable as part of HVTs, with a high enough volatility to simulate sulphur mustard in vapour exposure studies in decontamination units (Torngren et al., 1998).

MeS as a potential simulant for sulphur mustard has also been modelled to assess the similarity (Tanimoto coefficient) and dissimilarity (Euclidean Distance) according to molecular weight, solubility, vapour pressure and partition coefficients while also taking into account molecular parameters such as bond connectivity, stereochemistry, conformational variability and substructural fingerprints. When compared to distilled mustard, MeS has a Tanimoto coefficient of 0 (1 = identical, 0 = no similarity) and a Euclidean distance of 0.587 (0 = identical), which suggest that when mathematically modelled, MeS has very little similarity to sulphur mustard (Lavoie et al., 2011), which contrasts with other opinions in the literature (Bartelt‐Hunt et al., 2008).

Comparisons can be made between this review and a previous study (Bartelt‐Hunt et al., 2008), which identified MeS as one of the most suitable simulants for a range of parameters, while also being low enough in toxicity to be used in HVTs. The persistence of MeS is sufficient to analyse accurately the effective decontamination, while remaining volatile enough to allow for potential vapour‐related studies.

This review has reached the same conclusion as others regarding the use of malathion as the most suitable simulant for organophosphate compounds such as VX, due to the low dermal toxicity, high persistence and low vapour pressure of malathion. While malathion may be a more suitable simulant for the analysis of CWAs in which large scale, lengthy decontamination protocols are required, ethical issues are of concern.

When using a particular simulant for HVT of decontamination, it is important to use the minimal dose suitable to fulfil the requirements of the trial. The amount applied to volunteers should be enough to determine clear differences between consecutive decontamination interventions, while remaining low enough to be effectively removed. It is also important that the toxicity and dermal penetrations are taken into account when applying the dose. For trials in which metabolites are measured, the dose needs to be an appropriate concentration to detect the metabolites above potentially endogenous levels.

There are some limitations associated with this review. While every effort was made to conduct a systematic review of the literature, it remains possible, although remote, that some relevant studies were not considered. Furthermore, the relative toxicity values used to determine the most suitable simulants were obtained from different species of animals and cannot be accurately compared due to differences in biology and metabolism. Similarly, oral LD_50_s cannot be compared with neither dermal LD_50_s nor inhalation LC_50_s and vice versa, but due to the lack of coherent and harmonized data the most relevant relative toxicities have been included.

While in vivo and in vitro studies can be conducted with the use of TICs and CWAs or toxic simulants, decontamination trials involving human volunteers need to be conducted with a simulant suitable to apply to human skin and hair. This literature review identified 1055 articles and publications where 22 simulants were reported to have been used in in vitro, *in silico* and HVTs. From the 22 simulants reported, 17 were excluded by evaluating the toxicity data from MSDS sheets and additional resources.

The five remaining simulants (MeS, diethyl malonate, ethyl lactate, malathion and DCP) were further scrutinized for potential suitability for use in HVTs of emergency decontamination. These simulants were evaluated according to toxicity and physicochemical similarities to their equivalent toxic counterpart. MeS (simulant for sulphur mustard), diethyl malonate (simulant for soman) and malathion (simulant for VX or TICs) were identified as suitable CWA simulants for HVTs of emergency decontamination processes. This review provides a useful resource for the identification of CWA simulants suitable for use in HVTs of decontamination. Such trials can provide evidence‐based interventions for emergency decontamination during chemical incidents.

## Supporting information

Data S1. Supporting information item
