# Supplementary material for: Chemical warfare agent simulants for human volunteer trials of emergency decontamination: A systematic review
Source: J Appl Toxicol. 2017 Oct 9;38(1):113–21. doi: 10.1002/jat.3527 (PMC5725685; doi:10.1002/jat.3527)
Supplement: Supplementary file 1 — Data S1. Supporting information item [file JAT-38-113-s001.pdf]

Chemical warfare agent simulants for human volunteer trials of emergency decontamination: A systematic review

Thomas James[1,*], Stacey Wyke[1], Tim Marczylo[2], Sam Collins[1], Tom Gaulton[1], Kerry Foxall[2], Richard Amlôt[3] and Raquel Duarte-Davidson[1].

[1] Chemicals and Poisons, Centre for Radiation, Chemicals and Environmental Hazards (CRCE), Public Health England, Chilton, OX11 0RQ

[2] Toxicology, Centre for Radiation, Chemicals and Environmental Hazards (CRCE), Public Health England, Chilton, OX11 0RQ

[3] Emergency Response Department Science & Technology, Public Health England, Porton Down, Salisbury, Wiltshire, SP4 0JG, UK.

*Corresponding Author

Thomas James, Centre for Radiation, Chemicals and Environmental Hazards (CRCE),

Public Health England, Chilton, OX11 0RQ

tom.james@phe.gov.uk; Tel: +44 1235 825278

Supplementary material Table A

Literature Search Strings

Scopus

ALL (simulant*) AND ALL (decont*) AND ALL (skin OR hair) AND (ALL (non W/2 toxic) OR ALL (chemical W/2 warfare))
100 Document Results

Google Scholar
simulant* AND decon* AND (skin OR hair) AND (non-toxic) AND (chemical warfare)
214 results

Ovid Medline Search

1	exp Decontamination/	(3934)
2	Decont*.tw.	(9341)
3	Wet Decont*.tw.	(9)
4	Dry Decont*.tw.	(3)
5	1 or 2 or 3 or 4	(11078)
6	exp Chemical Warfare Agents/	(24856)
7	Simulant*.tw.	(1269)
8	non-toxic.tw.	(10870)
9	6 or 7 or 8	(36809)
10	Exp Fluorescent Dyes/	(100376)
11	Fluorescen*.tw.	(378104)
12	10 or 11	(427670)
**13**	**5 and 9 and 12**	**(11)**

Web of Science

# 1	TS=decontamination	15,911
#2	TS=decont*	18,836
#3	TS=simulant	2,710
#4	TS=chemical warfare agent	3,511
#5	TS=non-toxic	14,163
#6	#5 OR #4 OR #3 OR #2 OR #1	38,366
#7	TS=hair	81,012
#8	TS=skin	483,746
#9	TS=human trials	124,614
#10	TS=vivo	923,462
#11	#9 OR #8 OR #7	670,123
#12	#11 AND #6	1,976
**#13**	**#12 AND #10**	**265**

Supplementary Material - Table B: Simulants that failed to meet inclusion criteria when evaluated on toxicity.

**Omitted simulant**	**Rationale for elimination** (hazard statements).			
2-(chloroethyl) ethyl sulfide (CEES)<br>CAS 693-07-2	Acute Tox 3.<br>H301 Toxic if swallowed<br>H311 Toxic in contact with skin<br>H331 Toxic if inhaled	Skin Corr 1B.<br>H314 Causes severe skin burns and eye damage	Carc 1A.<br>H350 May cause cancer	
2-(chloroethyl) methyl sulfide (CEMS)<br>CAS 542-81-4	Acute Tox 3.<br>H311 Toxic in contact with skin<br>H331 Toxic if inhaled	Acute Tox 4.<br>H302 Harmful if swallowed	Skin Corr 1B.<br>H314 Causes severe skin burns and eye damage	Carc 1A.<br>H350 May cause cancer
2-(bromoethyl) phenyl sulfide (BEPS)<br>CAS 4837-01-8	Skin Irrit 2.<br>H315 Causes skin irritation	Eye Irrit 2A.<br>H319 Causes serious eye irritation		
2-(chloroethyl) phenyl sulfide (CEPS)<br>CAS 5535-49-9	Acute Tox 3.<br>H311 Toxic in contact with skin<br>H331 Toxic if inhaled	Skin Corr 1B.<br>H314 Causes severe skin burns and eye damage	Carc 1A.<br>H350 May cause cancer	
O,S-diethyl methylphosphonothioate (OSDEMP) CAS 6996-81-2	Acute Tox 4.<br>H302 Harmful if swallowed			
Dimethyl 4-nitrophenyl phosphate<br>CAS 950-35-6	Acute Tox 1.<br>H300 Fatal if swallowed			
Tetrahydrothiophene<br>CAS 110-01-0	Acute Tox 4.<br>H302 – Harmful if swallowed<br>H332 – Harmful if inhaled<br>H312 – Harmful in contact with skin	Skin Irrit 2.<br>H315 Causes skin irritation	Eye Irrit 2.<br>H319 Causes serious eye irritation	
Dimethyl methyl phosphonate<br>CAS 756-79-6	Eye Irrit 2A.<br>H319 Causes serious eye damage	Repr 2.<br>H361f Suspected of damaging fertility or the unborn child	Muta 1B.<br>May cause genetic defects	
Diisopropyl methylphosphonate (DIMP)<br>CAS 1445-75-6	Acute Tox 3.<br>H301 Toxic if swallowed<br>H311 Toxic in contact with skin<br>H331 Toxic if inhaled	Specific Target Organ Tox – single exposure 1.<br>H370 Causes damage to organs		
Dimethyl sulphide<br>CAS 75-18-3	Eye Irrit 2.<br>H319 Causes serious eye irritation			

Diethyl sulphide<br>CAS 352-93-2	Eye Irrit 2.<br>H319 Causes serious eye irritation	Skin Irrit. 2<br>H315 Causes skin irritation
Paraoxon<br>CAS 311-45-5	Acute Tox 1.<br>H330 Fatal if inhaled	Acute Tox 2.<br>H300 Fatal if swallowed<br>H310 Fatal in contact with skin
Parathion	Acute Tox 2.<br>H300 Fatal if swallowed<br>H330 Fatal if inhaled<br>H311 Toxic in contact with skin	Specific Target Organ Tox – Repeated Exposure 1.<br>H372 Causes damage to organs through prolonged or repeated exposure
Diethyl 4-nitrophenyl phosphate<br>CAS 950-35-6	Acute Tox 2.<br>H300 Fatal if swallowed	

Each compound has been presented with hazard (H) statements according to the Globally Harmonized System of Classification and Labelling of Chemicals (GHS) that explain the reason for omission. Tetrahydrothiophene is the only chemical that has a standardised classification across the European community through the European Chemicals Agency (ECHA), so data was sourced from Annex VI of the Classification, labelling and Packaging (CLP) regulation. All other compounds, while not harmoniously classified, have been subject to self-classification through the supplier of the chemical. This could be due to the fairly uncommon nature and specific usage of the simulants..
